# Which Is a Better Predictor of GFR Decline: 24-h Urine Protein or 24-h Protein–Creatinine Ratio? An Exploration of the MDRD Study Data

**DOI:** 10.3389/fneph.2021.797431

**Published:** 2022-01-27

**Authors:** Isabelle Ayoub, Haikady N. Nagaraja, Rima Kang, Brad Rovin, Udayan Bhatt

**Affiliations:** ^1^ Division of Nephrology, Department of Internal Medicine, The Ohio State University Wexner Medical Center, Columbus, OH, United States; ^2^ Division of Biostatistics, College of Public Health, The Ohio State University, Columbus, OH, United States

**Keywords:** proteinuria, 24h protein, 24h protein/creatinine, progression of kidney disease, measured GFR

## Abstract

**Background:**

Proteinuria is a known risk factor for progression of chronic kidney disease. Proteinuria magnitude can be estimated by measuring spot urine protein-to-creatinine ratio (least accurate), 24-h urine collection for protein (24 P), or 24-h protein–creatinine ratio (24 PCR). The MDRD study found that 24 P measured at baseline was the strongest single predictor of the rate of GFR decline during study follow-up. However, predictive powers of 24 P and 24 PCR have not been compared in the literature. The current study addresses this question using the MDRD cohort data.

**Methods:**

The study is a retrospective analysis of prospectively collected data from the MDRD cohort using simple and multiple regression models. Slope of measured GFR (mGFR) over time was used as the response and models that included baseline 24 PCR or 24 P were compared for the entire sample and for subgroups formed by restricting the values of 24-h creatinine and 24 P.

**Results:**

Log 24 P and Log 24 PCR correlated almost equally with mGFR slope. However, in simple linear regression models and multivariable linear regression models adjusting for age and sex, the model with 24 PCR had a higher *R* ^2^ than the corresponding one that had 24 P except for the subgroup 24 P < 1 g.

**Conclusion:**

We observe that 24 PCR may be a better marker of proteinuria magnitude in predicting decline in kidney function compared to 24 P in particular for patients with 24 P ≥ 1. This finding needs validation in prospective clinical trials.

## Introduction

Proteinuria is a known risk factor for progression of chronic kidney disease (1, 2). Proteinuria magnitude can be estimated by measuring a randomly collected spot urine protein-to-creatinine ratio (least accurate), 24-h urine collection for protein (24 P) or the protein-to-creatinine ratio from a 24-h urine collection (24 PCR). A random spot urine PCR cannot reliably assess 24 P or changes in 24 P (3). The time of the random urine collection, the degree of proteinuria, and the underlying cause of kidney disease affect the accuracy of random spot urine PCR (4, 5). In clinical trials, 24 P or 24 PCR are being used to quantify proteinuria as an entry criterion and for trial endpoints. For instance, the Supportive Versus Immunosuppressive Therapy for the Treatment of Progressive IgA Nephropathy (STOP IgAN) trial (6) used 24 P to determine inclusion but used 24 PCR to define study endpoints. Conversely, the Abatacept and Cyclophosphamide Combination Therapy for Lupus Nephritis (ACCESS) trial used 24 PCR for both study entry and endpoints (7). The Modification of Diet in Renal Disease (MDRD) study found that 24 P measured at baseline was an independent predictor of glomerular filtration rate (GFR) decline during study follow-up (8). Other studies in glomerular diseases have shown that 24 P, sustained proteinuria (24 P), and time-averaged proteinuria (24 P) after treatment were predictors of long-term GFR decline (9–13).

The advantage that 24 PCR has over 24 P is that it corrects for either over- or under-collection of an intended 24-h urine sample. However, as mentioned in several studies, a 24 P can be adjusted based on the measured:expected creatinine ratio in the intended 24-h sample (3, 14, 15). Unfortunately, this adjustment is not routinely applied in clinical trials and perhaps rarely in clinical practice. Whether 24 P or 24 PCR is the better predictor of GFR trajectory has not been reported. Our hypothesis is that 24 PCR is a better predictor of GFR decline than 24 P. To the best of our knowledge, the present work will be the first to assess whether 24 P or 24 PCR is a better predictor of GFR decline. The strength of this study is that GFR was measured directly and not estimated from serum creatinine levels.

## Methods

### Design

This is a retrospective analysis of prospectively collected data. Slopes of measured GFR (mGFR) over time as a function of baseline 24 PCR and 24 P were compared. The study was approved by The Ohio State University Institutional Review Board.

### Cohort

The study population included all patients from the MDRD trial who had a baseline 24-h urine protein and urine creatinine level, and an mGFR at baseline and at the end of the study. **Table 1** and **Supplementary Tables 1, 2** describe patient characteristics.

**Table 1:** Patient characteristics.

Baseline Patient Characteristics	*N* = 835
**Age (years)**	Mean (SD) 51.2 (12.36)
	Median (IQR) 52 (42; 62)
	*N* = 827 (missing = 8)
**Race/Ethnicity**	*N* (%)
** White**	703 (85)
** Black**	65 (8)
** Hispanic**	38 (5)
** Asian**	11 (1)
** Other**	10 (1)
	Total = 827 (missing = 8)
**Sex**	*N* (%)
** Men**	505 (61)
** Women**	322 (39)
	Total = 827 (missing = 8)
**Cause of CKD (see** **Supplementary Tables 1 and 2** **for more details)**	*N* (%)
** Polycystic kidney disease**	198 (24)
** HTN nephrosclerosis**	134 (16)
** Diabetic nephropathy**	24 (3)
** Glomerular diseases**	205 (25)
** Other**	215 (26)
	Total = 776 (missing = 59)
**HTN**	*N* (%)
** Yes**	694 (84)
** No**	133 (16)
	Total = 827 (missing = 8)
**DM**	*N* (%)
** Yes**	41 (5)
** No**	786 (95)
	Total = 827 (missing = 8)
mGFR ml/min*1.73 m^2^	Median (IQR) 31.44 (22.08; 42.65)
	*N* = 835
**24-h urine protein (g)**	Median (IQR) 0.32 (0.07; 1.47)
	*N* = 835
	≥1 g (*N* = 260)
	≥2 g (*N* = 161)
	≥3 g (*N* = 97)
**24 PCR (g/g)**	Median (IQR) 0.23 (0.05; 1.04)
	*N* = 835
**24-h urine creatinine (g)**	Median (IQR) 1.4 (1.1; 1.7)
	*N* = 835
**BMI kg/m^2^**	*N* (%)
** <18.5**	8 (0.9)
** ≥18.5–<25**	270 (32.7)
** ≥25–<30**	354 (43)
** ≥30**	193 (23.4)
	*N* = 825 (missing = 10)

### Baseline and Outcome Measures

GFR was measured as the renal clearance of ^125^I- iothalamate (16, 17). Baseline mGFR was the last mGFR in the baseline period of the MDRD study. 24 P and 24 PCR were measured from 24-h urine samples. The baseline 24 P and PCR were measured from the last 24-h urine collection in the baseline period of the MDRD study. Expected 24-h urine creatinine levels were calculated on 827 patients with available baseline age and weight and were compared with the measured 24-h urine creatinine levels to determine the completeness of the 24-h urine collections (15). A total of 827 patients (99.7%) had an intended 24-h urine that was ≥50% complete, thereby reliably estimating the PCR of a complete 24-h urine collection (3). 24-h P were not corrected as this is not routinely applied in clinical practice.

The primary goal of this study was to compare the rate of decline in kidney function in an individual according to 24 PCR versus 24-h P. The secondary goal was to examine the effect of age, sex, race, BMI, and baseline mGFR on proteinuria and decline in kidney function.

### Statistical Methods

Individual linear regression lines were fit to determine the rate of decline in GFR for each subject. Association between the GFR slope and baseline values of log (24 P) and log (24 PCR) were examined using Pearson correlation coefficient. Log scale for 24 P and 24 PCR was used to improve a normal fit to the data (4). Significance of correlations was established using *t*-tests, and to determine the equality of dependent correlations, Steiger’s test (1980, Eq. 14) was used (18). Scatter plot of 24 PCR versus 24 P was used to examine the variability in the 24 PCR as a function of 24 P that revealed a systematic sex effect (**Supplement 2**). Association between log (24 PCR) and log (24 P) and sex was examined using multiple linear regression (**Supplement 3**). Association between the GFR slope and sex, race/ethnicity, BMI, age, and baseline values of mGFR, 24 P, and 24 PCR was examined using analysis of variance or simple linear regression methods, as appropriate. Using the covariates with significant association, multiple regression models with and without interactions were developed to predict the slope of GFR decline. A four-factor model with sex, age, 24 P, and 24 PCR, and their interactions as predictors with all significant predictors, developed for the entire data set was checked for outliers and normality of residuals. After removing 11 outliers with studentized residuals outside (-4,4), this model was used to compare the predictive powers of baseline 24 P and 24 PCR for the entire data set, and for two subgroups: (a) subjects with baseline 24-h urine creatinine values ≤ 800 or ≥ 1,200 mg and (b) its subsets with baseline 24 P values of <1 g, ≥1 g, ≥2 g, and ≥3 g. The subgroup excluding 24-h urine creatinine values near 1,000 mg (1 g) was selected since the PCR value would have a denominator of 1 and therefore would strongly approximate the 24-h P value. This investigation was repeated for single- and three-factor sub-models that included 24 P or 24 PCR as predictors. Prediction equations based on various models were generated. Vuong’s test, implemented using the nonnest2 package in R (19), was used to compare non-nested three-factor models (20). All other statistical analyses were done using JMP 13 (SAS Institute, Cary, NC). Level of significance was set at 0.05 and no adjustments were made for multiple testing.

## Results

The final study population included 835 patients from the MDRD cohort that had baseline 24 P, 24 PCR, and an mGFR at baseline and at the end of the study. The mean duration of study follow-up was 2.2 years. There was a statistically significant correlation between GFR slope and log (24 P) (Pearson correlation, *r* = −0.2946, *p* < 0.0001), and between GFR slope and log (24 PCR) (*r* = −0.2929, *p* < 0.0001). Of these 835 patients, we then excluded 254 who had 24-h urine creatinine between 800 and 1,200 mg to eliminate concordance between 24 P and 24 PCR. In the excluded cohort, there was a near-perfect association between 24 PCR and 24 P and the association between these variables and GFR slope was identical (**Supplement 4**). For the sub-cohort of 581 patients, there was a statistically significant correlation between GFR slope and log (24 P) (*r* = −0.2677, *p* < 0.0001) and between GFR slope and log (24 PCR) (*r* = −0.2693, *p* < 0.0001). However, these correlations were not significantly different from each other (**Table 2**, third column). This result was consistent as well across the following subgroups: 24 P <1 g, ≥1 g, ≥2 g, and ≥3 g (**Table 2**). Nonetheless, in all subgroups except for 24 P <1 g, the correlation between GFR slope and log (24 PCR) was consistently higher than the one between GFR slope and log (24 P), and the difference in correlations increased as the proteinuria cutoff increased.

**Table 2:** Comparisons of pearson correlations (Corr) of variables with overlapping correlations.

Group	*N*	*r*_12_: Corr [mGFR slope, log(24 P)] (*p*-value*)	*r*_13_: Corr [mGFR slope, log(24 PCR)] (*p*-value*)	*p*-value (Null hypothesis: *r*_12_ = *r*_13_)**	Corr [log(24 P)], log[24 PCR)]
All subjects	835	−0.2946	−0.2929	0.7707	0.9845
		(<0.0001)	(<0.0001)		
A. Urine creatinine ≤800 or ≥1,200 mg	581	−0.2677	−0.2693	0.7974	0.9879
		(<0.0001)	(<0.0001)		
B. A. and Proteinuria <1 g	386	−0.1012	−0.0990	0.8581	0.9707
		(0.0469)	(0.0519)		
C. A. and Proteinuria ≥ 1 g	195	−0.2954	−0.3153	0.4715	0.9188
		(<0.0001)	(<0.0001)		
D. A. and Proteinuria ≥ 2 g	130	−0.3198	−0.3735	0.2245	0.8574
		(0.0002)	(<0.0001)		
E. A. and Proteinuria ≥ 3 g	78	−0.2097	−0.3023	0.2057	0.7831
		(0.0654)	(0.0071)		

*Based on the t-test for Correlation (r) = 0; ** Based on z-test, Steiger’s (18), Eq. (14), for equality of dependent overlapping correlations.

Using simple linear regression analysis, Model *R* ^2^ was consistently higher in model 1 (24 PCR) compared to model 1 (24 P) except for the subgroup 24 P <1 g where *R* ^2^ was higher in model 1 (24 P) (**Table 3A**). The associations between GFR slope and the variables sex, race/ethnicity, age, BMI, and baseline mGFR were examined individually. Only age (*r* = 0.09, *p* = 0.007) and sex [mean GFR slope (Female–Male) = 0.0875, *p* = 0.029] were also significantly associated with GFR slope. The variables age, sex, 24 PCR, 24 P, and the only 2 significant interactions (sex * 24 P and sex * 24 PCR) were all significant predictors of the full model 2 developed for the entire cohort (**Table 3B**). It is worth noting that in this full model that contains both 24 PCR and 24 P, the *p*-value of the slope estimate for 24 PCR was significant across all subgroups except for 24 P <1 g and ≥3 g and it was lower than that of 24 P, which was not significant in all subgroups. Furthermore, sex * 24 PCR interaction effect had smaller *p*-values than that of sex * 24 P in all cases (details not shown) and it was significant in more subgroups (**Table 3B**). Finally model 3 (24 PCR) that included 24 PCR, age, sex, and the interaction (sex * 24 PCR) was compared to model 3 (24 P) that included 24 P, age, sex, and the interaction (sex * 24 P) (**Table 3C**). Model *R* ^2^ was higher in model 3 (24 PCR) compared to model 3 (24 P) except for the subgroup 24 P <1 g where both *R* ^2^ were very small and similar. Although in the six different pairs of non-nested models considered in **Table 3C**, model *R* ^2^ in model 3 (24 PCR) and model 3 (24 P) are distinguishable except for subgroup 24 P<1 g, the Vuong test showed a significant superiority of model 3 (24 PCR) over model 3 (24 P) only for the sub-cohort (urine creatinine (≤800 mg, ≥1,200 mg) but not in the subgroups of proteinuria where the *p*-value was close to being significant (**Table 3C**). Regression equations for the entire cohort in models 1, 2, and 3 are summarized in **Supplementary Table 3**.

**Table 3A:** Simple linear regression models (24 PCR Model 1 and 24 P Model 1): Summary findings.

Group	*N*	24 PCR Model 1	24 P Model 1
		(no covariates)	(no covariates)
		*R^2^*, *p*-value	*R^2^*, *p*-value
All Subjects	824	17.19%, <0.0001	14.07%, <0.0001
A. Urine creatinine ≤800 or ≥1,200 mg	572	18.42%, <0.0001	13.86%, <0.0001
B. A. and Proteinuria <1 g	381	2.5%, 0.0018	2.75%, 0.0011
C. A. and Proteinuria≥ 1 g	191	17.88%, <0.0001	8.95%, <0.0001
D. A. and Proteinuria ≥ 2 g	126	23.50%, <0.0001	9.50%, 0.0004
E. A. and Proteinuria ≥ 3 g	75	24.82%, <0.0001	6.99%, 0.0219

**Table 3B:** Full multivariable linear regression model 2: Summary findings.

Group	*N*	Model 2 *R^2^*	Model 2
			*p*-values for main effects
			of PCR, P
All Subjects	816	20.30%	<0.0001, 0.0320^†^^
A. Urine creatinine ≤800, ≥1,200 mg	565	21.71%	<0.0001, 0.0782^†^^
B. A. and Proteinuria <1 g	376	7.48%	0.4839, 0.7536
C. A. and Proteinuria ≥ 1 g	189	23.31%	0.0096, 0.6257^†^
D. A. and Proteinuria ≥ 2 g	124	29.57%	0.0095, 0.7897^†^
E. A. and Proteinuria ≥ 3 g	74	31.93%	0.1050, 0.8027

Model 2 contains: (Main effects: sex, age, PCR, P; Interactions: sex * PCR, sex * P). All effects are significant with full data. ^†^Interaction effect of PCR and sex is significant. ^ Interaction effect of P and sex is significant.

**Table 3C:** Multivariable linear regression model 3: Summary findings.

Group^†^	PCR Model 3	P Model 3	*p*-value (Vuong tests: PCR Model 3 fits better than P Model 3)
	*R^2^*	*R^2^*	
	(*p*-value: PCR, Interaction)	(*p*-value: P, Interaction)	
All subjects	19.04%	16.41%	0.0591
(816)	(<0.0001, 0.0246)	(<0.0001, 0.0001)	
A. Urine creatinine ≤800 mg, ≥1200 mg	20.51%	16.48%	0.0458
(565)	(<0.0001, 0.0049)	(<0.0001, 0.0002)	
B. A. and Proteinuria <1 g	7.42%	7.21%	0.39
(376)	(0.0019, 0.9195)	(0.0066, 0.6115)	
C. A. and Protein ≥ 1 g (189)	22.46%	16.01%	0.0539
	(<0.0001, 0.0024)	(<0.0001, 0.0007)	
D. A. and Protein ≥ 2 g (124)	29.26%	21.02%	0.0669
	(<0.0001, 0.0029)	(<0.0001, 0.0002)	
E. A. and Protein ≥ 3 g (74)	31.57%	24.55%	0.157
	(0.0002, 0.0133)	(0.0002, 0.0019)	

^†^Sample sizes are the same as in **Table 3B**.

24 PCR Model 3 Predictors: sex, age, 24 PCR, sex * PCR.

24 P Model 3 Predictors: sex, age, 24 P, sex * P.

## Discussion

Proteinuria is an established clinical biomarker of prognosis in chronic kidney diseases (2). Nephrotic range proteinuria is arbitrarily defined as 24 P ≥ 3.5 g (21). At least in clinical trials, there is no consensus as to whether 24 P or 24 PCR represents a better criterion for inclusion and a better endpoint. Using the MDRD cohort, we found that 24 PCR might be a more favorable marker compared to 24 P in predicting decline in kidney function. Confounders and interactions will not be specifically discussed in detail although they are important; however, they are not directly relevant to the main goal of this work.

Log 24 P and Log 24 PCR correlated with each other and separately almost equally to mGFR slope. However, in simple linear regression models (24 PCR model 1 and 24 P model 1) and multivariable linear regression models adjusting for age and sex (24 PCR model 3 and 24 P model 3), the model that had 24 PCR had a higher *R* ^2^ than the corresponding one that had 24 P except for the subgroup 24 P <1 g. When both 24 PCR and 24 P were included in the same model 2, the *p*-values for the slope estimate of 24 PCR and its interaction were lower and more frequently significant compared to the 24 P ones. In addition, Vuong test in Model 3 showed that the model that had 24 PCR was a better fit than that of 24 P with statistical significance for the sub-cohort (24-h urine creatinine ≤800 mg or ≥1,200 mg). One way of thinking about the 24 PCR being a better predictor can be speculated as follows: all factors held constant, a 24 P adjusted for 24-h creatinine (24 PCR) is actually adjusting proteinuria for the size of the individual’s nephrons. For any given 24 P, 24 PCR can vary enormously from individual to individual. Filtered protein is known to induce tubulo-interstitial damage (22–26). It is plausible that for a given amount of filtered protein load, larger nephrons are less susceptible to damage than smaller nephrons given the difference in ratio of protein load to glomerular basement membrane surface area.

As mentioned earlier, in the sub-group 24 P <1, the model *R* ^2^ in both 24 P and 24 PCR model 1 and model 3 was lower and almost equal. On the other hand, *R* ^2^ in 24 PCR model 1 and 3 increased progressively in the sub-groups of proteinuria (≥1, 2, and 3) compared to the entire cohort and was higher than the corresponding *R* ^2^ in 24 P models 1 and 3. These observations suggest that for a proteinuric endpoint of <1 g/day, whether we use 24 P or 24 PCR is the same. However, for therapeutic decisions and especially if the 24 P ≥1, it may be more reasonable to use 24 PCR for a proteinuria threshold to avoid over- or under-treating. For simplicity and moving forward, we propose establishing 24 PCR as a standard for proteinuria entry criterion and as a marker for endpoint in clinical trials.

This study has several limitations. It is a retrospective analysis of the MDRD cohort. The cohort was not balanced in terms of cause of chronic kidney disease and did not include active immune-mediated kidney diseases with modest or marked proteinuria. It is also known that GFR progression in heterogeneous populations may not be linear. However, in spite of the limited MDRD individual level data, the use of linear regression models to estimate the slope of mGFR results in highly interpretable coefficient compared to more complex models. Although we attempted to adjust for the potential effect of relevant covariates such as age and sex, we acknowledge that other factors not included may still play a confounding role such as blood pressure profile and medications received throughout the study follow-up. The sample size in the proteinuria subgroups was small and likely affected the significance of the Vuong test for better model fitting. The difference between 24 P and 24 PCR as well as their potential clinical implications were not examined. This would have included eligibility for enrollment in clinical trials as well as standard of care management based on 24 PCR versus 24 P cutoff in a cohort of patients with active proteinuric glomerular diseases.

In summary, we observe that 24 PCR may be a better marker of proteinuria magnitude in predicting decline in kidney function compared to 24 P in particular for patients with 24 P ≥1. Certainly, this finding needs validation in prospective clinical trials and cannot be assumed to apply to active and non-active proteinuric glomerular diseases.

## Data Availability Statement

Publicly available datasets were analyzed in this study. This data can be found here: MDRD dataset.

## Ethics Statement

The studies involving human participants were reviewed and approved by The Ohio State University Institutional Review Board. Written informed consent for participation was not required for this study in accordance with the national legislation and the institutional requirements.

## Author Contributions

IA: Design, analysis and writing. HN: Data analysis and writing. RK: Writing and editing. BR: Editing. UB: Design, analysis and editing. All authors contributed to the article and approved the submitted version.

## Conflict of Interest

BR reports personal fees from Aurinia, Calliditas, ChemoCentryx, Retrophin, Novartis, Morphosys, EMD Serono, Bristol Myers Squibb, Janssen, Omeros, and AstraZeneca; non-financial support from Lupus Foundation of America; and grants from the National Institutes of Health (NIH), outside the submitted work.

The remaining authors declare that the research was conducted in the absence of any commercial or financial relationships that could be construed as a potential conflict of interest.

## Publisher’s Note

All claims expressed in this article are solely those of the authors and do not necessarily represent those of their affiliated organizations, or those of the publisher, the editors and the reviewers. Any product that may be evaluated in this article, or claim that may be made by its manufacturer, is not guaranteed or endorsed by the publisher.
