# Supplementary material for: Which Is a Better Predictor of GFR Decline: 24-h Urine Protein or 24-h Protein–Creatinine Ratio? An Exploration of the MDRD Study Data
Source: Front Nephrol. 2022 Jan 27;1:797431. doi: 10.3389/fneph.2021.797431 (PMC10479620; doi:10.3389/fneph.2021.797431)
Supplement: Supplementary file 1 [file DataSheet_1.pdf]

Supplement 1

Supplement Table 1. Etiology of Kidney disease diagnosis: evidence level, count and percentage

Level	N	%
1	243	31.1
2	539	68.9
Total	782	1
Missing	53	

1= Presumptive primary kidney diagnosis
2= Established kidney diagnosis by biopsy, serological studies or imaging

Supplement Table 2. Etiology of Kidney disease diagnosis, count and percentage

Diagnosis	N	%
Polycystic kidney disease	198	25.5
Hereditary nephritis	19	2.5
Analgesic nephropathy	11	1.4
Pyelonephritis	25	3.2
Other interstitial nephritis	36	4.7
Obstructive uropathy acquired	2	0.2
Obstructive uropathy congenital	5	0.6
Vesico-ureteral reflux	15	2.0
Urinary tract stones	6	0.8
Hypertensive nephrosclerosis	134	17.3
Diabetic nephropathy	24	3.1
Renal artery stenosis	1	0.1
Membranous nephropathy	16	2.1
Focal segmental glomerulosclerosis	70	9.0
Membranoproliferative glomerulosclerosis	19	2.4
Mesangial proliferative	7	0.9
CKD with proteinuria	39	5.0
Nephrotic syndrome without biopsy	6	0.8
Single kidney	26	3.3
IgA nephropathy	44	5.7
Other glomerulonephritis	43	5.5
Other	30	3.9
Total	776	
N Missing	59	

Supplement table 3. Summary of regression equations and predictions for the entire cohort for different models

**Model (N)**	**Group**	**Regression equation** EstimateGFR(Slope)=	**Predicted slope for average subject^**
24 PCR Model 1 (824)	All	- 0.1949 - 0.1515*PCR	-0.2297 (F or M)
24 P Model 1 (824)	All	- 0.2027 - 0.1014*P	-0.2351 (F or M)
Full model 2 (816)	Females	- 0.3717 + 0.0040* age – 0.3574*PCR +0.1922*P	-0.1884
	Males	-0.4389+ 0.0040* age - 0.0539*PCR- 0.0449*P	-0.2617
24 PCR Model 3 (816)	Females	-0.3399 + 0.0037*age - 0.1751*PCR	-0.1915
	Males	-0.4239 +0.0037*age-0.1233*PCR	-0.2636
24 P Model 3 (816)	Females	-0.3148 + 0.0030*age -0.1544*P	-0.2112
	Males	-0.3852+ 0.0030*age – 0.0781*P	-0.2572

^Age 51years (mean), 24 PCR =0.23, 24 P=0.32 (medians)

Supplement 2. Scatter plot of 24 PCR versus 24 P revealed a systematic sex effect. The blue circles correspond to males and the red ones to females

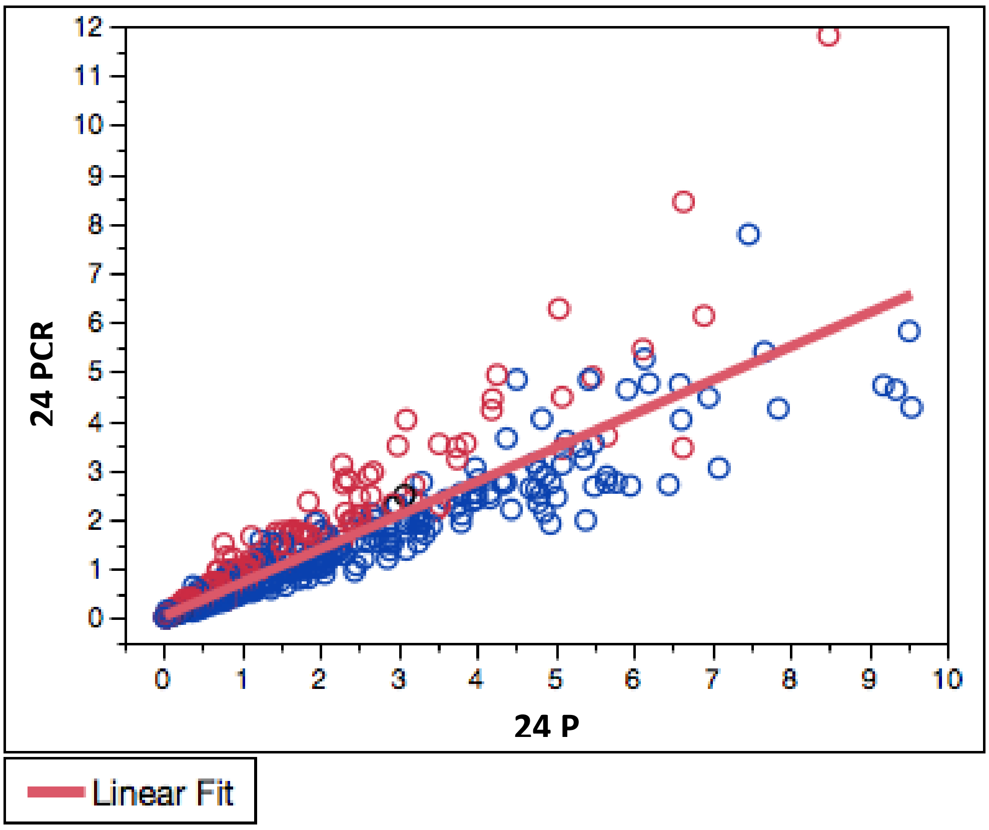

Supplement 3. Regression Plot and summary measures of fit

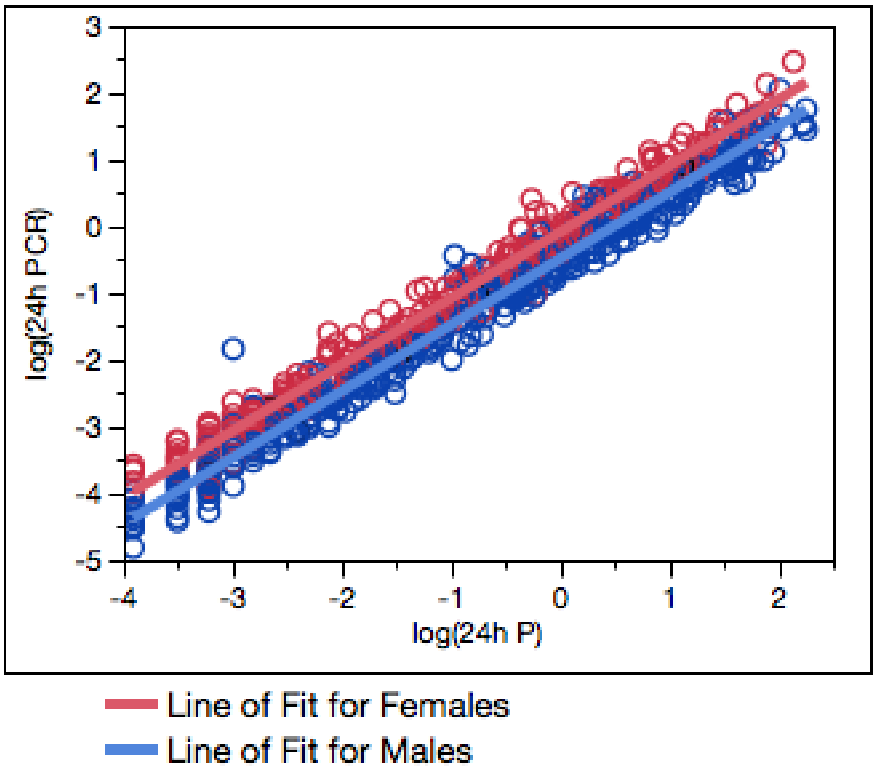

Summary of Fit

RSquare	0.982169
RSquare Adj	0.982126
Root Mean Square Error	0.224255
Mean of Response	-1.41994
Observations (or Sum Wgts)	827

Analysis of Variance

Source	DF	Sum of Squares	Mean Square	F Ratio
Model	2	2282.5867	1141.29	22694.18
Error	824	41.4391	0.05029	**Prob > F**
C. Total	826	2324.0258		<.0001*

Parameter Estimates

Term	Estimate	Std Error	t Ratio	Prob>|t|
Intercept	-0.278002	0.009646	-28.82	<.0001*
log(24h P)	0.993857	0.004666	213.01	<.0001*
Sex[F]	0.1990015	0.00807	24.66	<.0001*

Note: The high R^2 (98%) is due to the fact that most of the variability in log(PCR) = log(P) –log(Cr), is due to the variability in log(P).

Supplement 4. Spearman correlations in the excluded cohort

Variable	by Variable	Spearman rank correlation
24 PCR	24 P	0.9973
GFR(Slope)	24 P	-0.3324
GFR(Slope)	24 PCR	-0.3283
